# Effects of Socioeconomic Status, Parent–Child Relationship, and Learning Motivation on Reading Ability

**DOI:** 10.3389/fpsyg.2018.01297

**Published:** 2018-07-25

**Authors:** Qishan Chen, Yurou Kong, Wenyang Gao, Lei Mo

**Affiliations:** Guangdong Key Laboratory of Mental Health and Cognitive Science, Center for Studies of Psychological Application, School of Psychology, South China Normal University, Guangzhou, China

**Keywords:** socioeconomic status, reading ability, parent–child relationship, learning motivation, moderated mediation model

## Abstract

Against the background of Chinese culture, we investigated the relationship between family socioeconomic status (SES) and children’s reading ability. Participants included 2294 middle-school students in grade 8. SES was measured by parents’ education level, parents’ occupational prestige, and family property, and children’s reading ability was estimated with item response theory. In addition, we adopted an 8-item parent–child relationship scale and a 22-item learning motivation scale that included four dimensions. We examined whether the parent–child relationship mediated the relationship between family SES and reading ability and whether this was moderated by learning motivation. The results indicated that the parent–child relationship played a mediating role in the relationship between SES and reading ability. This relationship was moderated by students’ learning motivation. The direct effects of SES on reading ability at high, medium, and low levels of learning motivation were 0.24, 0.32, and 0.40, respectively.

## Introduction

Reading, the process of acquiring meaning from text, is one of the most complex and unique cognitive activities of humans. Reading ability can have a significant influence on both the academic achievement and further personal development of students (Espin and Deno, 1993; Herbers et al., 2012; Reed et al., 2017). Therefore, it is necessary to investigate the factors that influence students’ reading ability and to explore the possible mechanisms of these factors. Numerous studies have shown that personal characteristics, family socioeconomic status (SES), teachers, and school characteristics are key factors affecting students’ reading ability and academic achievement (Sirin, 2005; Stanovich, 2009; Law, 2011; Chiu and Chow, 2015). Among them, SES is one of the most common factors and is the most discussed.

### SES and Academic Achievement

Socioeconomic status reflects and is measured by the social and economic status of family members. People generally believe that there is a strong and stable correlation between SES and children’s academic achievement and cognitive development. However, the conclusions from studies are inconsistent (Bradley and Corwyn, 2002; Lareau, 2011). Many researchers have found that family background factors can explain most of the variance in students’ academic achievement and play a more important role than schools (Arnold and Doctoroff, 2003; Reardon, 2011; Berkowitz et al., 2017; Lawson and Farah, 2017). The positive correlation between SES and academic achievement persists from childhood to adolescence and is consistent across races (Mpofu and Van de Vijver, 2000; Wössmann, 2005; Aikens and Barbarin, 2008; Caro et al., 2009; Kieffer, 2012; Ren and Xin, 2013). However, some studies have shown that SES has little to no relevance for academic achievement (Rech and Stevens, 1996; Seyfried, 1998; Ripple and Luthar, 2000). A meta-analysis conducted by White (1982) of almost 200 studies showed a positive correlation between SES and academic achievement, with an average of 0.35 and a median of 0.25. Another meta-analysis performed by Sirin (2005) of more than 70 studies published from 1990 to 2000 found that there was not a high correlation between SES and academic achievement. The average was 0.29, and the median was 0.24. These meta-analyses both showed that the relationship was moderated by variables including the personal characteristics of students, the definition and measuring method of SES, and the measuring index of academic achievement.

Students’ personal characteristics, such as grade, age, race, or ethnicity, are seen as important moderator variables. Several longitudinal studies found that the lower children’s SES is, the worse their academic achievement, and this relation was consistent across ages of children (Walker et al., 1994; Pungello et al., 1996). However, both meta-analyses showed that this relation decreased gradually over time (White, 1982; Sirin, 2005).

The measuring method of SES is also a vital moderator variable. Scarr and Weinberg (1978) found that parents’ education level could be as predictive as other factors for children’s academic achievement. However, Mercy and Steelman (1982) argued that although different indicators of SES (family income and parents’ education level) could all predict children’s intelligence score, the mother’s educational attainment acted as a better predictor than the father’s. It is clear that different components of SES could influence different aspects of specific cognitive skills or academic achievement (Parcel and Menaghan, 1990). An index of status characteristics proposed by Warner et al. (1949) that includes four dimensions – occupation, income, accommodation, and living region – was widely adopted in the early stage of this research field. With increasing academic interest in the role of parents’ education level and occupation, a two-factor index of social position has also been used by several researchers (Hollingshead and Redlich, 1958). The socioeconomic index (SEI) designed by Duncan (1961) estimates SES based on the income and education level of each occupation. The Michigan State Department of Education directly defines SES as having three dimensions: family income, parents’ education level, and parents’ occupation; this definition has been used extensively in numerous studies (Gottfried, 1985; Hauser, 1994; Bornstein and Bradley, 2014). Therefore, we adopted this definition and used parents’ education level, occupational prestige, and income level to measure family SES.

Parents’ education level can be measured using scales of both diploma attainment and schooling years. Compared with data on schooling years, diploma data are relatively easy to collect because many students, especially those in lower grades, may not know or be able to calculate the number of years their parents have attended school. This may lead to missing or artificial data. To maintain accuracy in the measurement of parents’ education level, we collected diploma data from students.

The prestige of an occupation can be measured based directly on the occupational classification. However, this method tends to leave out new occupations and fails to reflect the class differentiation within one occupation. For example, the Occupational Classification Pandect of the People’s Republic of China excludes many new occupations, such as seasonal migrant worker and freelancer, and business owners’ social status and prestige vary significantly based on the scale of their enterprises. Another method is to require students to describe the occupation and job category and then have coders categorize the occupations and assign them values according to the International Standard Classification of Occupations (ISCO), which was formulated by the International Labor Organization. Despite consuming more money and time, the second method can achieve more accuracy and higher validity than simply gathering occupation information from students. Given that the Chinese occupational classification is incompatible with the ISCO, the Chinese Occupational Prestige Measuring Index compiled by Li (2005) was adopted in this study. There are 81 occupations that received a score ranged from 9.73 to 90.15 and was classified into seven prestige levels according to the scores.

The measurement of income, which seems easy, is difficult to conduct in practical situations such as this one. The most direct method is to ask students or their parents to report monthly or annual income. However, many people are reluctant to disclose the real amount of their income, especially in Chinese culture, where income is widely considered a private matter. In addition, hidden income and income mobility might undermine data authenticity. Another measuring method that has been widely used in multiple studies is to ask students to report their family property. The Family Affluence Scale (FAS, Currie et al., 1997) measures family wealth with this method and asks students the following questions: Do you have your own bedroom? Does your family own a car, a truck or a van? How many computers are there in your family? How often has your family traveled during the past 12 months? Trends in International Mathematics and Scientific Studies (TIMSS) investigates family education resources based on access to a dictionary, the child’s own desk, a computer, and the number of books (Mullis et al., 2005). The Programme for International Student Assessment (PISA) requires students to report the type and amount of electrical equipment in their home, the number of cars in the family, housing conditions, bathing conditions, and so forth. This method was also used in empirical research with a Chinese cultural background (Ren and Xin, 2013). In this paper, we adopted the second method. With the aim to better represent or easily distinguish the family economic conditions, taking the practical situation in China into consideration, we chose equipment such as TV, refrigerator, home ownership, car, washing machine, air conditioner, and computers as indicators of the index.

The measuring index of academic achievement functions as another moderator variable. In the educational context, academic achievement can be measured not only by a general index such as GPA or IQ but also by a specific index such as language and math scores. White’s (1982) meta-analysis suggested that the strongest correlations between SES and different indexes were those for IQ (0.40), GPA (0.26), reading performance (0.31), and math performance (0.25).

We proposed an operational definition and measuring framework of reading ability based on well-known pre-existing measuring programs (i.e., PISA, PIRLS, and NAEP) in combination with the definition and analysis in China’s *Full-time Compulsory Education Curriculum Standard Chinese* (Mullis et al., 2009; National Assessment Governing Board, 2009; Organization for Economic Co-operation and Development, 2012). Given that the form and the content of reading materials are two important influencing factors, we set three different conditions: reading literary texts, reading continuous information texts, and reading non-continuous information texts. We investigated three different reading abilities reflected during the reading procedure: retrieving and inferencing, integrating and interpreting, and evaluating and reflecting.

The form of reading material refers to how a text is organized, that is, continuous text or non-continuous text. The content of reading material refers to the type of information transmitted, that is, literary text or informational text. Therefore, combining the two forms and two types of content would result in four pairs. However, in view of the practical feature of reading material and middle-school students’ reading practice, literary texts are mostly continuous. Accordingly, three reading situations were adopted in this study.

The first condition was reading literary texts; the test material included fairy tales, fables, fiction, or prose. The second condition was reading continuous informational texts; the test material included introductions and explanatory texts such as expositions, scientific essays, and argumentations. The third condition was reading non-continuous informational texts; the test material mainly included practical texts such as graphs, tables, and advertisements.

In this study, 55% of reading materials are literary texts, 30% are continuous informational texts, and 15% are non-continuous informational texts, which was set based on the Chinese Full-time Compulsory Education Curriculum Standard.

Three kinds of reading ability were examined: retrieving and inferencing, integrating and interpreting, and evaluating and reflecting. Retrieving and inferencing involves retrieving explicit information and making simple inferences from it. Integrating and interpreting involves forming an overall perception and initial summary of the article and then inferring and explaining the implicit information within it. Evaluating and reflecting requires readers, with pertinent background information, to think critically regarding the content and form of the reading material.

By far, there are a number of research have discussed the relationship between SES and reading ability in both Chinese and western cultural background (Hoff, 2003; Noble et al., 2006; Rowe and Goldin-Meadow, 2009; Zhang et al., 2013; Wen et al., 2016; Chow et al., 2017; Pan et al., 2017; Su et al., 2017). However, they paid less attention to the internal mechanism of the relationship. Additionally, there are some deficiencies in the measurement of SES and reading ability in these studies. The purpose of the present study was to investigate the relation between family SES and students’ reading ability while controlling for the variables addressed by White (1982) and Sirin (2005). To achieve this goal, we adopted an SES index suited to the Chinese context and estimated reading ability using the item response theory (IRT) technique. We examined a moderated mediation model that includes parent–child relationship and students’ learning motivation.

### The Influence of Family SES on the Parent–Child Relationship and Children’s Reading Ability

Family SES plays a crucial role in children’s reading ability development. Many studies have made discoveries regarding the relationship between SES and reading ability (Hoff, 2003; Noble et al., 2006; Rowe and Goldin-Meadow, 2009). A lot of research has highlighted the importance of SES in children’s reading ability in the Chinese cultural context (Zhang et al., 2013; Wen et al., 2016; Chow et al., 2017; Pan et al., 2017; Su et al., 2017) For example, Zhang et al. (2013) examined the relations among SES, vocabulary, and reading with 262 children who had diverse SES backgrounds and were followed from ages 4 to 9 in Beijing, China. They found that SES contributed to variance in phonological skills and vocabulary in the early developmental stages. A longitudinal study conducted by Su et al. (2017) investigated the predictive power of early family factors for children’s reading literacy at the end of primary school with 262 Chinese children. The results indicated that family SES and parent–child reading engagement were associated with literacy skills. Wen et al. (2016) examined the influence mechanism of family SES on student reading ability in China based on a questionnaire and a reading test completed by 574 eighth grade students from two medium-sized counties. These results also verified the influence of family SES on children’s reading ability.

It is often considered that the influence from SES on children’s academic achievement tends to be indirect, and SES can initiate changes in some other factors (Bradley and Corwyn, 2002). The mediating variables of child, family, and school characteristics may be substantial channels for the influence of SES on academic achievement (Sirin, 2005). In addition to material and social resources, non-monetary factors provided by the family are important for children’s academic achievement (Kim and Rohner, 2002; Tsui, 2005). SES influences academic achievement and cognitive development through a series of family environment variables such as parents’ educational expectations, parenting ideas and behaviors, and the parent–child relationship (Bradley et al., 2001; Yeung et al., 2002). Based on an integration of results from studies of preschool, primary, and grade school children, Hess and Holloway (1984) identified that the relation between parents and children is one of the important variables linking socioeconomic factors to school achievement.

As discussed previously, the relations between SES and children’s reading ability are complex and parent–child relationship may be characterized as a “bridge” between them. Family SES is a reflection of the social and economic resources that parents can provide (Bradley and Corwyn, 2002). It can affect parents’ cognitive and reactive modes in relation to society and family members (Duncan et al., 1994). According to the family stress model, parents in low SES families face more financial pressure and emotional exhaustion, which are associated with low income and self-efficacy (Conger and Donnellan, 2007). This may cause parents to use negative, unkind strategies to get along with their children and result in an undesirable parent–child relationship (McLoyd, 1990; Conger et al., 1994). Previous research has demonstrated that SES has a positive correlation with parent–child connectedness (*r* = 0.27; Clark and Ladd, 2000). The undesirable relationship may deprive children of advantageous psychological circumstances that benefit their cognitive development. By contrast, parents in high SES families have much more time, energy and knowledge about education, and they are inclined to express more warmth and affection in order to cultivate a favorable parent–child relationship (Kraus et al., 2012; Dixson et al., 2017). Family relationships are important to Chinese students’ cognitive development and academic performance. Positive parent–child interactions or relationships have been found to be correlated with good reading ability development (Chan, 1981). Lau and Leung (1992) found that better relationships with parents and school peers lead to higher academic performance, including higher class rank, higher final exam scores, and higher scores in Chinese, English, mathematics, physical education, and music. This is because in a favorable relationship, parents devote more attention to educating their children and show more enthusiasm, which can provide children emotional support and in turn enhance their academic performance and reading ability. In this study, we would test whether parent–child relationship mediate the relation between SES and children’s reading ability

### The Influence of Learning Motivation

The influence of SES on academic achievement is not the same for all children. Moderating variables, including demographic variables such as grade, age, and race, and external supporting variables such as family, school, and community, is most often discussed (White, 1982; Bradley and Corwyn, 2002; Sirin, 2005). However, researchers have paid less attention to students’ internal characteristic variables when discussing the moderators of the direct effect of SES on academic achievement. Our study focuses on students’ learning motivation, which reflects the extent of challenge, engagement, intrinsic motivation, and extrinsic motivation and examines it in a moderated mediation model.

From the academic resilience perspective (Arellano and Padilla, 1996), although academic risk factors can block academic development, resilience factors such as learning motivation help children overcome risk factors (Alfaro et al., 2009). Some evidence has shown that learning motivation plays a moderating role in the relation between academic performance and certain personal variables, especially intrinsic motivation, which occurs when individuals engage in activities based on interests and enjoyment (Ryan and Deci, 2000; Spinath and Steinmayr, 2012). The abovementioned personal variables also include learning experience, test anxiety, and psychological distress (Salami, 2008; Ning and Downing, 2012; Khalaila, 2015). Another study found that intrinsic motivation explained more variance in the reading performance of low ability readers than that of high-ability readers (Logan et al., 2011). The results of this study indicated that children with low reading skill who had higher intrinsic motivation tended to persevere more in developing their abilities, but those who had lower intrinsic motivation tended more to abandon the effort to learn. Likewise, low SES is also an undesirable condition, and motivation might moderate the relationship between SES and reading ability because the role of motivation may be more crucial for low SES children than for high SES children. Recently, Kim et al. (2017, 2018) conducted a series of longitudinal studies to examine why young adults who attended eighth or ninth grade in Dalian City, China, in 1999 believed that their poorer middle-school classmates were more likely to do well academically than their wealthier classmates. Based on interviews with 48 respondents, they found that students of poorer parents were more motivated to gain upward mobility through academic achievement. There is an old saying in China: “Children from poor families take up responsibilities early.” Students from poor families grow up in a relatively difficult environment. They may want to change their current situation more urgently than students who are better off, and they may think that it will be easier to do so if they study harder and do better at school. In other words, family SES influences individual success differently according to the motivation. Children with similar family SES may not have the same academic achievement. We proposed that such discrepancies may be caused by the different levels of learning motivation among children. We assumed that for students with strong motivation, the influence of SES on reading ability is weakened. However, for students with weak motivation, the influence of SES through the mediating variable is strengthened.

The purpose of the present study was to examine whether parent–child relationship mediate the relation between SES and children’s reading ability and whether this mediating relationship can be moderated by students’ learning motivation. Based on the previous literature (e.g., Hess and Holloway, 1984; Bradley and Corwyn, 2002; Spinath and Steinmayr, 2012; Zhang et al., 2013; Wen et al., 2016; Kim et al., 2017), we propose the following hypotheses: (1) family SES positively relates to children’s reading ability, (2) parent–child relationship mediates the positive relationship between SES and reading ability, and (3) learning motivation moderates the influence of SES on reading ability.

## Materials and Methods

### Participants

We used a cluster random sampling method to recruit 2294 middle-school students in grades 8 from 11 schools in Beijing and Guangzhou to participate in our study. Of this total, 1091 were from Beijing (male = 497, female = 594), and 1203 were from Guangzhou (male = 609, female = 583, unreported = 11).

The present study was approved by the Research Ethics Committee, South China Normal University. All participants provided their oral informed consent before completing the measures. The data were collected and analyzed anonymously.

### Variables Measured

#### Family SES

Socioeconomic status was defined as having three dimensions: family income, parents’ education level, and parents’ occupational prestige. This definition has been widely used in the academic research, and the present study used it to measure family SES. Parents’ education level was reported by students and divided into five levels. The coders defined parents’ occupation type based on the students’ description of their parents’ occupation and job category, and then, they assigned values to the rank of the occupation type using Li’s (2005) Chinese Occupational Prestige Measuring Index. Student reports of the amount of family property, which included purchased houses, cars, air conditioners, computers, etc., were used to measure family income. The factor analysis showed that these indexes belonged to a factor, and the accumulated variance contribution rate was 44.04%. The factor score obtained was taken as the raw score of family property. Ultimately, we transformed the raw score of the three indexes into a standard score and summed them into composite SES points.

#### Reading Ability

Participants’ reading ability was estimated by IRT, which is a modern psychometric approach that has been successfully applied in psychological and educational research in recent years (Rouse et al., 1999; Chernyshenko et al., 2001; Junker and Sijtsma, 2001; Silver et al., 2001). IRT has a number of advantages over classical test theory (CTT). One of the major advantages is that the estimates of test item parameters (e.g., difficulty) and examinee ability are independent of one another (Hambleton et al., 1991). In CTT, item parameters depend on a representative sample from the target population (Embretson, 1996). For example, item difficulty is defined in terms of the scores obtained by examinees taking a test. When examinees have low ability, the test will appear to be difficult, and when examinees have high ability, the test will appear to be easy. By contrast, in IRT, examinee ability and test difficulty are described by monotonically increasing functions called item characteristic curves (ICC). These curves describe how changes in ability level relate to changes in the probability of a correct response, and they are determined by one or more item and ability parameters. As a result, an IRT-based test yields unbiased estimates of item properties and provides valuable insight into the role of test difficulty in reading scores because the researchers developing reading tests generally do not have ready access to representative samples. Because of its psychometric properties, an IRT-based comprehension test may provide a better measure of comprehension than tests used in prior research.

We proposed the measuring framework of reading ability and developed an original item bank accordingly. The original item bank, containing 38 texts and 228 test items, was designed and developed by an expert panel. After a pilot test conducted with 1203 grade 8 subjects recruited in Guangzhou City, another group of experts retained 25 texts and 130 questions. Then, the remaining 130 items were distributed by following a balanced incomplete block design (see **Table 1**). Ten booklets, each containing 26 items, were designed so that any participant could complete a booklet in less than 60 min. After the testing, participants’ responses were collected, cleaned, input, and analyzed based on the two-parameter model (2-PLM) of IRT. As a result, some items were removed from the item bank. The reliability and validity of the item bank were examined (the mean Cronbach’s alpha coefficient of the ten booklets was 0.73), and the discrimination and difficulty parameters of the items and the ability parameters of students were estimated. Finally, 108 valid questions were retained. IRT analysis was conducted for all remaining 108 items. Using the 2PL model, item discrimination parameters *a* and item difficulty parameters *b* were estimated. Item difficulty *b* ranged from -2.89 to 3.50 (*M* = -0.91, *SD* = 1.25). Item discrimination *a* ranged from 0.18 to 1.17 (*M* = 0.55, *SD* = 0.23). Students’ ability levels ranged from -3.27 to 1.79 (*M* = -0.07, *SD* = 0.81).

**Table 1:** Calibration design of reading ability module administration.

Block	Booklet										No. of records	No. of items
	1	2	3	4	5	6	7	8	9	10		
1											460	15
2											461	11
3											464	15
4											457	11
5											453	15
6											461	11
7											462	15
8											455	11
9											455	15
10											460	11
N of records	232	229	235	222	231	230	232	223	232	228	2294	
N of items	26	26	26	26	26	26	26	26	26	26		130

#### Parent–Child Relationship

We adopted the Parent–child Relationship Scale (Hair et al., 2005), which has eight items measured on a 4-point Likert scale, to measure participants’ relationship with their parents and their parents’ attitudes and expectations. Example items are “My parents are proud of me,” “My parents will encourage and comfort me when I encounter some troubles,” “I’m satisfied with the relationship between me and my parents,” and “My parents will accompany me if there is an important activity.” The scale was translated from the original, back-translated, and adjusted for cultural adaptation. First, we conducted exploratory factor analysis (EFA) with half of the participants (*n* = 1147). The results showed that the scale had a one-dimensional structure. Eight indexes had high loading on one factor and explained 48.46% of the total variance. Then, we performed confirmatory factor analysis (CFA) with the other half of the participants. The factor loadings of every item were between 0.50 and 0.80. The goodness-of-fit indexes were χ^2^ = 352.83, NNFI = 0.93, CFI = 0.95, and SRMR = 0.06. Cronbach’s alpha coefficient of this scale was 0.86.

#### Learning Motivation

We adopted the Learning Motivation Scale (Cheng et al., 2013), which has 22 items measured on a 5-point Likert scale, to measure participants’ learning motivation. This scale contains four dimensions: challenge, engagement, intrinsic motivation, and extrinsic motivation. Example items include “I care greatly about how others think about my school performance,” “I like to attempt to solve complex problems in schoolwork,” and “I don’t care about scores and rewards as long as I’m doing what I like to do.” EFA with half of the participants (*n* = 1147) showed that the scale had four dimensions that explained 51.53% of the total variance. CFA with the other half of the participants showed that the factor loadings of every item were between 0.45 and 0.85, and the goodness-of-fit indexes were χ^2^ = 1436.98, NNFI = 0.92, CFI = 0.93, and SRMR = 0.08. Cronbach’s alpha coefficient of the four dimensions and the whole scale was 0.84, 0.83, 0.72, 0.66, and 0.87.

### Measurements and Data Analysis

We adopted a paper-pencil test and took the class as a group. Each participant received a pack of test questions, which included two parts of the reading test (a total of 26 questions) and a background questionnaire. The time allotted for the test was divided into two periods with a break between them. We used a balancing technique: half of the participants did the reading test first, and the other half did the background questionnaires first. The participants were allocated to these conditions randomly.

We used BILOG, SPSS Version 21.0, LISREL, and Mplus Version 7.4 to analyze the data. First, we used the expectation–maximization algorithm to handle missing data in SPSS. Then, we tested hypothetical models using path analysis in Mplus with maximum likelihood estimation. At the same time, we used bias-corrected bootstrapping procedures with 2000 bootstrap samples to compute the point estimate value and 95% bias-corrected confidence intervals (Preacher and Hayes, 2008).

## Results

### Descriptive Statistics

The results of the descriptive statistics are shown in **Table 2**. We can see that family SES, the parent–child relationship, and learning motivation were all positively correlated with reading ability. The reading scores of males were significantly lower than those of females. Thus, we controlled for the gender factor in the following model test to decrease the spurious effect. Here, we conducted an independent-samples *T* test to compare the mean differences between students from Beijing and students from Guangzhou on all variables. No significant differences (*p* > 0.05) were observed.

**Table 2:** Descriptive statistics (*N* = 2294).

Variables	1	2	3	4	5
(1) Gender^a^	1				
(2) Reading ability	–0.20^∗∗^	1			
(3) SES	–0.04	0.35^∗∗^	1		
(4) Parent–child relationship	–0.07^∗∗^	0.18^∗∗^	0.18^∗∗^	1	
(5) Learning motivation	–0.03	0.23^∗∗^	0.19^∗∗^	0.37^∗∗^	1
*M*	0.48	–0.05	0.35	2.93	3.65
*SD*	–	0.81	1.75	0.63	0.54

*^a^0 = girl, 1 = boy (the mean value of gender represents the proportion of males in the sample); ^∗^*p* < 0.05, ^∗∗^*p* < 0.01, ^∗∗∗^*p* < 0.001.*

### The Effects of SES, Parent–Child Relationship, and Learning Motivation on Reading Ability

According to the test method of the moderated mediation model (Baron and Kenny, 1986; Wen et al., 2006; Preacher et al., 2007; Wen and Ye, 2014), we first tested whether the direct path between SES and reading ability was moderated by learning motivation. The model (Model 1) was a saturated model. Its fit was acceptable in a simple regression model without considering latent variables. The *R*^2^ of reading ability was 0.19. The result (see **Table 3**) showed that both SES (*b* = 0.33, *t* = 16.94, *p* < 0.001) and learning motivation (*b* = 0.15, *t* = 7.28, *p* < 0.001) were significantly related to reading ability. The interaction of SES and learning motivation was significantly related to reading ability (*b* = -0.08, *t* = -3.64, *p* < 0.001). Learning motivation played a moderating role between SES and reading ability.

**Table 3:** Parameter estimates of research models.

	Model 1		Model 3			
	Equation (DV: reading ability)		Equation 1 (DV: relationship)		Equation 2 (DV: reading ability)	
	*b*	*t*	*b*	*t*	*b*	*t*
Gender	–0.18	–9.57^∗∗∗^	–	–	–0.18	–9.39^∗∗∗^
SES	0.33	16.94^∗∗∗^	0.12	6.08^∗∗∗^	0.32	16.54^∗∗∗^
Motivation	0.15	7.28^∗∗∗^	0.34	13.05^∗∗∗^	0.13	5.96^∗∗∗^
SES × motivation	–0.08	–3.64^∗∗∗^	–	–	–0.08	–3.55^∗∗∗^
Relationship	–	–	–	–	0.06	2.86^∗∗^

*DV, dependent variable; ^∗^*p* < 0.05, ^∗∗^*p* < 0.01, ^∗∗∗^*p* < 0.001.*

Second, based on Model 1, we tested the moderation effect of learning motivation on the first stage (i.e., from SES to parent–child relationship) and the second stage (i.e., from parent–child relationship to reading ability). The *R*^2^ of the parent–child relationship was 0.14, and the *R*^2^ of reading ability was 0.19. The model (Model 2) fit was acceptable (CFI = 0.98, TLI = 0.91, RMSEA = 0.05, SRMR = 0.01). The interaction effect between SES and learning motivation on the parent–child relationship was statistically non-significant (*b* = -0.03, *t* = -1.42, *p* > 0.05). The interaction effect between the parent–child relationship and learning motivation on reading ability also statistically non-significant (*b* = -0.01, *t* = -0.44, *p* > 0.05). The interaction effect between SES and learning motivation on reading ability was, however, statistically significant (*b* = -0.08, *t* = -3.24, *p* < 0.01). The results indicated that learning motivation did not have a moderation effect between SES and reading ability on the first stage or the second stage.

**FIGURE 1:**
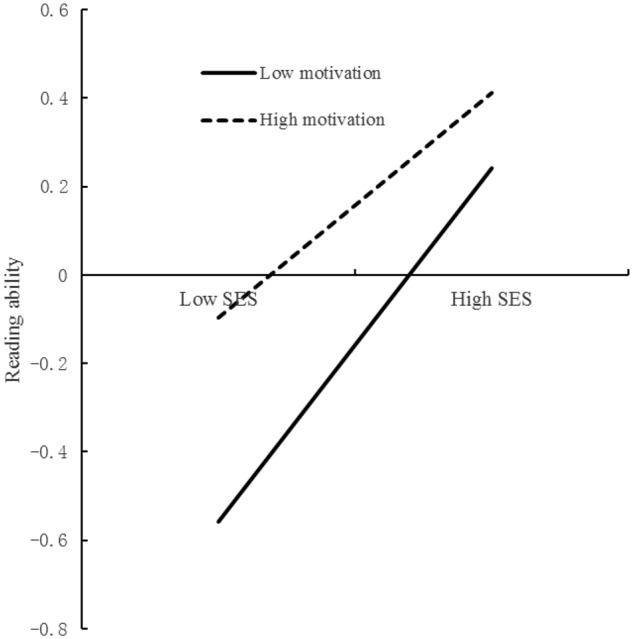
Learning motivation as a moderator of the direct relationship between SES and reading ability.

Finally, based on Model 2, we removed the interaction effect of learning motivation on the first stage and the second stage from the model. That is, we considered only the moderation effect of learning motivation on the direct effect. Consequently, the fit indexes of the new model (Model 3) were CFI = 0.99, TLI = 0.96, RMSEA = 0.04, and SRMR = 0.01. The *R*^2^ of the parent–child relationship was 0.14. The *R*^2^ of reading ability was 0.19. The results (**Tables 3, 4**) showed that SES (*b* = 0.12, *t* = 6.08, *p* < 0.001) was significantly related to the parent–child relationship. SES (*b* = 0.32, *t* = 16.54, *p* < 0.001), learning motivation (*b* = 0.13, *t* = 5.96, *p* < 0.001), the parent–child relationship (*b* = 0.06, *t* = 2.86, *p* < 0.01), and the interaction between SES and learning motivation (*b* = -0.08, *t* = -3.55, *p* < 0.001) were significantly related to reading ability. The mediation effect of the parent–child relationship was 0.01 (*t* = 2.58, *p* < 0.05, 95% CI [0.002, 0.012]). The direct effects of SES on reading ability differed according to the change in the learning motivation level. The direct effects of SES on reading ability at high (Mean + 1 *SD*), medium (Mean), and low levels (Mean - 1 *SD*) of learning motivation were 0.24 (*t* = 9.20, *p* < 0.001, 95% CI [0.19, 0.29]), 0.32 (*t* = 14.09, *p* < 0.001, 95% CI [0.27, 0.36]), and 0.40 (*t* = 10.92, *p* < 0.001, 95% CI [0.32, 0.47]), respectively. The results of simple slope test (Dearing and Hamilton, 2006) showed that the slope of high motivation was higher than that for low motivation (**Figure 1**). These findings revealed that the effect of SES on reading ability decreased as learning motivation increased.

**Table 4:** Direct effect, indirect effect, and total effect for Model 3.

	Direct effect *c*′	Indirect effect *ab*	Total effect *ab* + c′
			
SES to reading ability	0.32^∗∗∗^	0.01^∗^	0.33^∗∗∗^
Relationship to reading ability	0.06^∗∗^		0.06^∗∗^
Motivation as moderator			
High	0.24^∗∗∗^	0.01^∗^	0.25^∗∗∗^
Medium	0.32^∗∗∗^	0.01^∗^	0.33^∗∗∗^
Low	0.40^∗∗∗^	0.01^∗^	0.41^∗∗∗^

*^∗^*p* < 0.05, ^∗∗^*p* < 0.01, ^∗∗∗^*p* < 0.001.*

In conclusion, the parent–child relationship is the mediating variable between family SES and children’s reading ability. Learning motivation is the moderating variable of relationship between SES and reading ability.

## Discussion

The relationship between family SES and academic performance has always been an important issue in sociology, pedagogy, and psychology. With social and economic development and the improvement of research methods, more and more research has begun to pay attention to the mediator and moderator variables between SES and academic performance (Bradley and Corwyn, 2002; Sirin, 2005). The present study used eighth grade students from a Chinese cultural background as subjects to explore parents’ education level and professional prestige and family property as indicators of SES and reading ability, as estimated by IRT techniques, and to explore the influential mechanism of SES on reading ability. The results showed that the effect of SES on reading ability is mediated by the parent–child relationship, and this effect is moderated by students’ learning motivation.

### Family SES and Reading Ability

We used IRT to estimate reading ability instead of CTT. The potential advantages of utilizing IRT analysis in item and scale development include greater flexibility in selecting items from an existing item bank that can be tailored to the objectives of a particular research investigation (Fraley et al., 2000; Runge et al., 2018). By using IRT, we measured reading ability through participants’ responses on the test items. The estimation of participants’ reading ability with IRT depends not on specific test questions but instead on the response mode of participants (Embretson and Reise, 2013).

For the measuring method of SES, this study kept to the international conventions while simultaneously making the measurement culturally appropriate. The international occupation codes did not fit the Chinese condition because occupational classifications contain social identity implications; thus, we referred to Li’s (2005) Chinese Occupational Prestige Measuring Index. Moreover, instead of asking students about their parents’ income directly, we required them to report their family property, which included durable lifestyle goods that developed countries value and basic living conditions that developing countries value.

The existing research about the relationship between SES and academic achievement has not reached an agreement, and it contains considerable controversy. Studies have measured SES by different methods, and the effect factors of academic achievement are quite complicated; thus, it is not strange that different studies can draw different or even opposite conclusions. The present study found that the correlation coefficient of SES and reading ability was 0.35, which is quite similar to that in the meta-analyses conducted by White (1982) and Sirin (2005). We also found that the direct effect of SES on reading ability occupied a larger percentage of the total effect than the indirect effect. It is thus clear that SES has an effect on reading ability.

Given the results of this study, we can conclude that family SES does have a correlation with students’ reading ability. The higher the parents’ education level, occupational prestige and income are, the higher the children’s reading ability, and *vice versa*. The positive link between SES and children’s achievement is well established (White, 1982; McLoyd, 1998; Sirin, 2005). There is a relation between poverty and low SES for a range of negative child outcomes, including low IQ, educational attainment and achievement, and increased social–emotional problems. However, this relation is quite complex because the different components of SES impact reading ability in different ways (Bradley and Corwyn, 2002). Parental education is an important index of SES, and it is indeed an important and significant unique predictor of child educational achievement (Duncan and Brooks-Gunn, 1999; Davis-Kean, 2005). Parents who are not well educated may not have enough ability or emphasis for providing tutorship for their children’s academic attainment. This may cause children’s academic difficulty to accumulate increasingly over time.

With regard to occupation, low occupation status or prestige generally indicates heavy physical labor, long working hours, low wages, and unstable working opportunities (with a relatively high probability of being laid off). This may force parents to expend time and energy that would otherwise be directed toward supporting their children’s study. Previous research has shown that parents’ occupational prestige is related to their involvement and engagement activities with their children, which in turn are positively related to children’s achievement (Marsiglio, 1991; Hill et al., 2004).

With regard to income, families with low income may not be able to provide necessary living goods such as a house, a study area, or a computer and other supplements such as extracurricular books, newspapers, and magazines for children. In recent years, studies in cognitive neuroscience have revealed the relationship between family income and children’s academic performance. Income is logarithmically associated with brain surface area. Research found that among children from lower income families, small differences in income were associated with relatively large differences in brain surface area, whereas among children from higher income families, similar income increments were associated with smaller differences in surface area. These relationships were most prominent in regions supporting executive functions, language, and reading (Noble et al., 2015). In other words, income is most strongly related to brain structure and reading among the most disadvantaged children.

### The Effects of Parent–Child Relationship and Learning Motivation

This study showed that family SES influenced reading ability not only directly but also indirectly through the parent–child relationship. More interestingly, we also found that the direct effect was moderated by students’ learning motivation, which means that the effect of SES on reading ability can differ depending on students’ learning motivation.

Socioeconomic status can indirectly influence children’s reading performance through the parent–child relationship established by parents’ speech and behaviors. Within this process, the parent–child relationship is an important form of externalized SES. A harmonious parent–child relationship is an indispensable component of healthy physical, mental, and cognitive development for children, and it is also a non-negligible factor for promoting children’s reading ability (Jeynes, 2003, 2007). Compared to parents with low education levels, those with high education levels provide more assistance and tutorship directly, and more importantly, they can provide assistance indirectly through a better parent–child relationship. They can do so by presenting a positive attitude and expressing educational expectations toward their children. Generally, parents with higher education levels know more about proper parenting styles and have more approaches for addressing difficulties in their relationships with their children. This ability can create a warm and harmonious parent–child relationship and, consequently, promote children’s academic performance. Bergin’s (2001) research revealed a significant relationship between the affective quality of the parent–child relationship and the child’s attitude toward reading as well as the child’s reading fluency. The Chinese phrase “children from a scholarly family” emphasizes the importance of the atmosphere fostered by the education level of parents and other family members for children’s academic achievement (Wen et al., 2016). With regard to the indirect effect of occupation and income on reading ability, parents with low SES often have more negative emotions, such as dissatisfaction and unhappiness, and experience more financial pressure. In such circumstances, they are more likely to take their anger out on their children and to discipline them by maltreatment in their rearing methods. As a result, children may feel aggrieved and dissatisfied, and their academic achievement may be affected.

The mediation effect of the parent–child relationship tells us that parents should not hold the simple view that providing sufficient material conditions for their children is enough for improving their academic performance. By contrast, a positive parent–child relationship and family atmosphere should also be built based on material conditions and educational investment.

We found that students’ learning motivation restrained the direct effect of the parent–child relationship on reading ability. The moderating effect of learning motivation revealed the complexity of the effect of SES on reading ability. Although the effect of SES on academic achievement was confirmed, in the real world, we can find examples of children in low SES families who achieve academic success and children in high SES families who fail in their academic performance. The reason for this phenomenon is that initiative factors such as learning motivation moderate the effect of SES on academic achievement. Children in low SES families or with undesirable parent–child relationships may lack opportunities to obtain material resources, and they may be faced with stressful life events as well as a passive family atmosphere. If they have strong learning motivation, they may overcome these unfavorable effects through active study attitudes and good learning habits. Thus, learning motivation can enhance the ability of children to cope with the adversity caused by low SES. As for children with high SES, although they may have more study resources or better academic support, they may face academic failure if their learning motivation is low.

The results of this study and those of Kim et al., 2018 mutually verify and support one another. Kim et al. (2018) drew on a survey of 503 respondents and found that children from poorer families performed better academically than those from wealthier families. Wealthier children were more likely than poorer children to lack motivation.

### Practical Implications and Future Research

Considering the direct effect of SES on reading ability, the government should provide better conditions for promoting the academic success of students by introducing a series of measures such as increasing the investment in less developed areas, remitting the tuition of destitute families, and offering scholarships for specific families.

In the light of the indirect effect of SES on reading ability through the parent–child relationship, parents should pay more attention to family education. The education, occupation and income of parents cannot be changed in a short time, but education attitude and parent–child relationships are comparatively easy to change. Parents should provide support and assistance to their children’s academic life through building a better family atmosphere.

As for the moderating effect of learning motivation, importance should be attached to the effect of students’ subjective initiative in removing the negative influence of family SES. School education and family education can arouse and maintain the learning motivation of children and encourage them to overcome the effects of harmful factors.

This study found significant relevance between family SES and students’ reading ability. However, we cannot understand this result in a simple and absolute way. First, we analyzed data only at the individual level, but the relation between SES and reading ability may vary based on higher level variables such as classes and schools. Second, all variables in this study were analyzed as observed variables. The results may be more accurate if potential variables were used in considering the measurement error. Finally, this is a cross-sectional study that cannot draw any conclusions about cause and effect. In future research, a longitudinal study may provide stronger evidence on this problem. To sum up, continued research should further refine the variables based on previous work and combine new statistical methods such as hierarchical linear modeling (HLM) and structural equation modeling (SEM).

## Author Contributions

QC contributed to developing the theoretical framework, data analysis, organization, and overall writing of the manuscript. LM contributed to developing the theoretical framework, editing and organization of the paper, as well as the overall design. YK and WG contributed to the design, data analysis, and editing of the manuscript.

## Conflict of Interest Statement

The authors declare that the research was conducted in the absence of any commercial or financial relationships that could be construed as a potential conflict of interest.
